# A Hybrid Energy Equating Game for Energy Management in the Internet of Underwater Things

**DOI:** 10.3390/s19102351

**Published:** 2019-05-22

**Authors:** Gulnaz Ahmed, Xi Zhao, Mian Muhammad Sadiq Fareed

**Affiliations:** 1 School of Management, Xi’an Jiaotong University, Xi’an 710049, China; gulnaz@mail.xjtu.edu.cn; 2 School of Electronic and Information Engineering, Xi’an Jiaotong University, Xi’an 710049, China; sadiqfareed@mail.xjtu.edu.cn

**Keywords:** Internet of Things, Internet of Underwater Things, Underwater Sensor Network, underwater monitoring, cluster-based architecture, best forwarder selection

## Abstract

The Internet of Underwater Things (IoUT) is an evolving class of Internet of Things and it is considered the basic unit for the development of smart cities. To support the idea of IoUT, an Underwater Sensor Network (USN) has emerged as a potential technology that has attractive and updated applications for underwater environment monitoring. In such networks, route selection and cluster-head management are still challenging. As the optimal routes always lead to congestion and longer delays while the cluster-head mismanagement leads to ending the USN lifespan earlier. In this paper, we propose a cooperative clustering game that is based upon energy heterogeneity and a penalty mechanism to deal with the cluster head mismanagement issue. Then, we use a non-cooperative evolutionary game for the best relay selection; the results prove that this utility function is the most suitable solution for the relay selection and its strategy selection converges to Nash Equilibrium. The proposed framework is compared with recent schemes using different quality measures and we found that our proposed framework performs favorably against the existing schemes for all of the evaluation metrics.

## 1. Introduction

The Internet of Underwater Things (IoUTs) is regarded as a technological revolution of communication and computing and is defined as a network of smart inter-connected underwater objects [1,2,3]. To support the idea of IoUT, Underwater Sensor Networks (USNs) are considered an emerging technology, which enables us to monitor the underwater environment, underwater exploration and disaster prevention in real-time [4,5]. The characteristics of USNs differ from territorial sensor networks in many ways such as transmission media, transmission range, propagation speed, and transmission rate. Most of the recent studies take into account problems like the lack of bandwidth, end-to-end delay and reliability, while a few of them consider dynamic network topology [1,6]. However, energy limitation is another point of concern for IoUT as the recharging cost of the Field Nodes (FNs) batteries deployed in the underwater environment is very high. Only this limited energy goal can be achieved by designing a protocol, which successfully reduces the number of transmissions and selects a reliable and congestion-free route for data delivery to minimize network energy consumption.

Recently, Game Theory (GT) is emerging as an influential tool to solve and investigate the energy management, Cluster Head (CH) selection and routing decisions issues. GT is widely applied in IoUTs algorithms to predict the behavior of FNs and to manage the FNs in USN under critical situations [7,8]. A two-level classical and evolutionary GT is applied to improve the uneven energy consumption for route and CH selection [8]. These two-levels are wedge-level and node-level, which are used to balance the energy at CH and node-level, respectively. Each FN is regarded as a rational decision-maker in USN and individually plays its smart game. Each player selfishly makes its decisions, which depends on its own interests. GT is very helpful in finding an equilibrium solution from the available strategies and very suitable for making decisions for the FNs. Each FN can freely make its own decisions for rendering its services as CH/FN or it has a choice to adopt a route because there is no incentive to deviate from the selected strategy [7]. However, achieving an equilibrium strategy and maximizing the payoff for each FN is not completely considered to date.

A territory game theoretical clustering protocol is discussed in Reference [9]; the entire sensing field is distributed into four equivalent regions like A, B, C and D. The multi-hop communication is used to forward the data from FNs to the Surface Sink (SS). The FNs in the closer regions have a higher probability to be selected as CHs. The authors tried to optimize the number of CHs from the neighboring region through an evolutionary game. This strategy slightly improves the performance by distributing the energy load on the entire network; however, due to the CH selection limit, the network lifetime is affected badly in dense and large-scale networks. Clustered routing for selfish sensors is discussed in Reference [10], the GT is applied to investigate the behavior of the CHs. As the CHs spend much more energy compared to the FNs, so the FN with low-energy can only render their services as a normal node. However, if each FN declines to be the CH, then limited nodes offer their services as CH. Consequently, these limited selected CHs cannot properly convey the data of all the FNs in the network to the SS, which leads to ending the network lifetime earlier.

An algorithm is designed in Reference [11], to tackle the issue that all the FNs in the Network Sensing Field (NSF) can exchange control packets that are not feasible for practical applications. The FNs in Reference [11] are only restricted to play a greedy selfish game with their local neighbors and only a restricted number of CHs are chosen from a certain territory. However, the utility function and a payoff function for adopting different strategies are not defined properly. To improve the criteria of CHs selection in Reference [11], a hybrid game theory-based distributed algorithm is defined in Reference [12]. The burden on CHs increases when the member nodes are at a longer distance from the SS, so the authors consider the node degree and the node’s distance from the SS when describing the payoff function.

**Figure 1:**
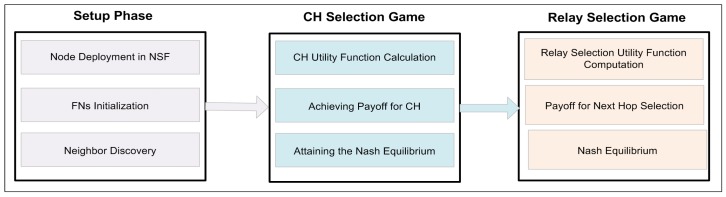
The flowchart of the proposed framework.

To overcome the issues of the previously designed schemes, we introduce a novel Hybrid Energy Equating Game (HEEG), which increases the network lifetime by reducing the redundant transmissions over the link while ensuring 99.9% data delivery at the SS. The spatiotemporal multi-cast and dynamic CH selections are capable of easily adjusting the floated nodes due to water current movements across the network. The data-aggregation hierarchy is simple and the data-forwarding routes are congestion free, which also helps to improve the network’s lifetime. In our proposed framework, there is no need to employ all the nodes for the sensing and collecting activities. Only the selected nodes from the different directions are chosen to perform the sensing as shown in Figure 1. Consequently, the proposed framework proves to be very energy-efficient with less end-to-end delay and high delivery ratio as compared to the existing schemes. The main contributions of our proposed framework are summarized as:

- We propose a clustering game to divide the FNs into different clusters by using the social correlation between them, which considers both energy and payoff function.
- We design a non-cooperative evolutionary game-based strategy for the best relay selection, which depends upon the set of available hops and strategy space for the players.
- We also prove that the next-hop strategy in the relay selection game is not invaded by any other mixed strategies. At each next hop selection, the nodes can modify their strategy until they reach a stable state.
- We performed extensive simulations to evaluate the proposed approach and found that the sensor node’s life relies completely on the number of transmissions, by avoiding the redundant transmission and controlling the auxiliary information so it does not circulate inside the network, the lifetime of the network can increase by 50–60%.

To design HEEG, we considered all the practical aspects and found it suitable for the hardware implementation system. However, the following limitations may occur in a real-time implementation:

- As the proposed model is redundancy-aware, sometimes the normal data in the overlapping region is considered as redundant and deleted.
- In the proposed model, only selected nodes from the different directions are chosen to perform the sensing activity. So, sometimes the information from a particular region may be missed due to this selection criterion.
- The proposed model supports the large-scale WSNs; however, the proposed model does not perform persuasively for very large-scale WSNs.

The remainder of this paper is organized in the following way: We discuss the current literature on cluster-based schemes for IoUTs in Section 2; in Section 3, the details of energy consumption and the signal-to-noise-ratio calculation models are briefly discussed; in Section 4, each stage of the proposed model, such as the cluster head selection game and the relay selection game, are discussed in detail; in Section 5, the evaluation metrics and comparisons are described to examine our proposed model; and finally, the conclusion is drawn in Section 6.

## 2. Related Works

Different approaches [13,14] have been designed to save the energy of the sensor nodes. Among these approaches, the cluster-based schemes are more efficient and save the energy of the network by reducing the number of transmissions. Instead of forwarding the data individually, a head node is chosen for forwarding the sensed information of all the Selected Member Nodes (SMNs) [15]. This sensed data is forwarded through a direct communication link or a cooperative communication link from the member nodes towards the SS. The cooperative communication links are preferred over the direct communication links when the data is forwarded at a longer distance. The role of the head node revolves among all the member nodes. The head node performs some extra duties like data collection and data fusion [13,15,16]. In performing such extra duties, the nodes consume some extra energy as compared to the other member nodes [17,18,19]. However, the poor head node selection criteria, oversize clusters, and redundant data due to overpopulated clusters, can deplete the battery of the head node much earlier than expected.

DEEP [14] is a multiple-hop based routing algorithm in which the collision probability between any two transmissions is analyzed and evaluated. In this protocol, the nodes are deployed randomly instead of a grid topology. An ad-hoc network is created using Autonomous Underwater Vehicles (AUVs) to monitor the underwater environment and the authors named the protocol the AUV-based Data Delivery Protocol (ADDP) [20]. Only the specific area where an event takes place can be monitored by AUVs and it is only possible if the AUVs behave in a cooperative manner. The AUV holding data starts sending control messages to its neighbors to create a communication link, the AUV arriving first will possibly establish a connection to forward the data. However, every time all the neighboring AUVs start moving to establish a link for conveying the data, it increases the overall operational cost of the network. A Path Reliability-Aware Data Delivery (PRADD) is defined in Reference [21] for large-scale networks. The FNs are deployed through the anchor and FNs convey the data through an accumulate-and-forward manner to the ferry. The next hop is only selected based on the coverage probability and reliability of the links. However, the FNs deployed closer to the SS are always busy forwarding the data of their predecessors. So, these FNs deplete their batteries earlier than the distant nodes.

Recently, some Mobile Sink (MS) or AUV-based approaches have been discussed in Reference [16,20]. In these AUV-based approaches, the AUV travels in a predefined trajectory called a tour path for complete coverage of the network. The AUV stops on tour points to collect the data from the FNs. These schemes are effective for small networks and efficiently reduce energy consumption. However, for a many layers network, these schemes are ineffective due to the long AUV trajectories, which increase both latency in data-gathering and the operational costs. The coverage problem for a many layers network is handled in Reference [22,23,24] using multiple-AUVs, where each AUV travels in a separate trajectory and cooperates with others to cover the whole network. However, the mobility of AUVs and the changing underwater conditions affect the communication between neighboring AUVs and the other nodes.

An evolutionary game was designed in Reference [25] to analyze the dynamic and cooperative performance of selfish FNs in a critical situation. The packets are repeatedly forwarded in two different modes to learn about the neighbor’s behavior and to improve the overall network performance. These two modes are the deterministic mode and the random mode. The deterministic mode investigates the selfish FNs behavior for the normal strategies while the later mode is applied to guessing the best possible strategies. Another GT based framework is modeled for distributed and adaptive routing [26]; the designed model collects the correlated information and incorporates the correlation information between the FNs. This game-theoretic approach is specifically designed to reduce the energy consumption on available routes by finding the best possible path.

The proposed model differs from current approaches in the following three ways:

- The cooperative clustering game of the proposed scheme is based upon the energy heterogeneity and a penalty mechanism in the payoff function, so, only those nodes offer their service as CHs which have higher energy and the maximum payoff. As a result of this solution, we achieve a tradeoff between providing data forwarding services and saving energy information delivery to the member nodes.
- The proposed model CH selection game is dynamic, so, the floated nodes due to water current movement at the end of a round can be easily adjusted as a member and can forward the data through the new CH, while the floated node during the transmission phase can request the new CH for conveying its data to the SS.
- The non-cooperative evolutionary game for the next hop selection of HEEG is most suitable for clustering architecture and we also prove that this utility function is the most suitable solution for the forwarder selection. Furthermore, this next hop strategy is not invaded by any other mixed strategies at each next hop selection; the nodes can modify their strategy until they reached a stable state.

## 3. Preliminaries

In this section, we discuss the details of energy consumption and the Signal-to-Noise-Ratio (SNR) calculation models.

### Energy Consumption

According to the first order radio energy model [27], in order to attain a suitable SNR in transmitting a *k*-bit message over a distance *d*, the energy spent by the radio is given by:

(1) $$\begin{array}{c} C_{tx}\left(k,d\right)=\left\{\begin{array}{cc} E_{elec}k+kE_{fs}d^{2} & \;ifd<d_{0}\\ E_{elec}k+kE_{amp}d^{4} & \;ifd\geq d_{0} \end{array}\right. \end{array}$$

where, $E_{elec}$ is the energy consumed per bit to run the transmitter or the receiver circuit, $E_{amp}d^{4}$ and $E_{fs}d^{2}$ represent the transmit amplifier coefficient of multi-path and free space models, and *d* represents the sender to the receiver distance. In case $d\geq d_{0}$, the transmit amplify coefficient of the multi-path model $E_{amp}d^{4}$ is used. Otherwise, the free space amplify coefficient $E_{fs}d^{2}$ is utilized. Furthermore, we considered a symmetric channel to transmit and receive the information. When the node receives this message, it expends $E_{Rx}\left(k\right)$ energy as:

(2) $$\begin{array}{c} C_{rx}\left(k\right)=kE_{elec} \end{array}$$

In the acoustic environment, the signal attenuation does not depend on the link distance *d* and equally considers the frequency *f*. Hence, an SNR for a signal which is generated under the frequency *f*, with unit transmission power and low bandwidth is given as ρ(d,f). The acoustic signal equation is defined in Reference [27,28,29] for an acoustic channel with a distance *d* between the source and destination at a signaling frequency f(KHz) and spreading coefficient *S* as:

(3) $$\begin{array}{c} A\left(d,f\right)=A_{o}\left[a{\left(f\right)}^{d}\right]d^{S} \end{array}$$

where, $A_{o}$ denotes the unit normalization constant, *S* is a spreading factor the value of which can be different depending upon the propagation environment as:

1. S = 1 for shallow water environment
2. S = 1.5 for practical environment
3. S = 3 for the deep water environment

The absorption co-efficient a(f) is taken in dB/Km as described by using the Thorp formula [30,31] as:

(4) $$\begin{array}{c} 10log\left(a\left(f\right)\right)=\left(0.11f^{2}\right)\times \frac{1}{1+f^{2}}+\left(44f^{2}\right)\times \frac{1}{4200+f}+2.75f^{2}\times 10^{-4}+0.003\;\;\;\;if\left(f>0.4\right) \end{array}$$

(5) $$\begin{array}{c} 10log\left(a\left(f\right)\right)=0.002+\left(0.11f\right)\times \frac{1}{1+f}+0.0011f\;\;\;\;if\left(f<0.4\right) \end{array}$$

Now the energy expenditure for an FN to transmit a *k* bit message to another FN at a distance *d* with the frequency *f* can be computed as:

(6) $$\begin{array}{c} C_{tx}\left(k,d\right)=\left\{\begin{array}{cc} E_{elec}k+ka{\left(f\right)}^{d}d^{2} & \;ifd<d_{0}\\ E_{elec}k+ka{\left(f\right)}^{d}d^{4} & \;ifd\geq d_{0} \end{array}\right. \end{array}$$

## 4. Hybrid Energy Efficient Clustering Game

To explain the operation of our framework, we divide its working into rounds (time steps). Then, every round is further divided into four steps, such as: (1) network setup; (2) CH selection game; (3) relay selection game; and (4) the data transmission. The network initialization and the network settling phases provide a way for uniformly distributing the energy and data load all across the network over and above help in keeping the network stable. At the same time, the last two steps help with cutting down the network energy by best route selection. The detailed information about HEEG framework is explained in the next subsections.

### 4.1. Network Architecture

In our proposed framework, the N number of FNs are uniformly installed in the NSF, the SS is supposed to be outside the sensing area. As we are totally unaware of the inside situation, random node distribution is an effective strategy. In this framework, we utilize the following random distribution method to install the FNs in the NSF:

(7) $$\begin{array}{c} P\left(N(A)=K\right)=\frac{\left({\lambda |A|}^{K}\exp^{-\lambda |A|}\right)}{K!} \end{array}$$

where, λ and |A| are the FNs density and area of the NSF, respectively. In this framework, the FNs sense the information in the installed vicinity and transfer the collected information through a CH to the SS. Thus, in this framework, three types of sensors are taking part such as FNs, CHs and the SS. Suppose that N and CHs signify the set of FNs and CHs, respectively. Where, $N=\{j|\;j\geq 1\;⋀\;j\;\leq \;j_{\max}\}$ and $j_{max}$ is the maximum number of FNs. $CHs=\{CH|CH\geq 1⋀CH\leq CH_{\max}\}$, where $CH_{max}$ is the maximum number of CHs. All the FNs, CHs and the SS are independent of making connections with each other. From this discussion, we have the following connectivity expression:

(8) $$\begin{array}{c} P_{\left(a,b\right)}=\left\{\begin{array}{cc} 1 & If\;a\;establishes\;a\;connection\;with\;b\\ 0 & Otherwise \end{array}\right. \end{array}$$

The a,b∈N, and a≠b. Here, b is either a member node or a CH. If b is a CH, then a is a member node. The quality of links is directly related to the received signal strength and the distance. Before discussing the operations of our proposed framework, we are making some assumptions as follows:

- The SS is an enriched resources device and it is equipped with both acoustic and radio modems. The radio modem is used for communication to the final processing center or offshore sink, while the acoustic modem is engaged in communicating with the CHs.
- The CHs are only relaying (working as a forwarder) the data of SMNs in their cluster and are not taking part in any sensing activity.
- The depth of the deployed FNs is different, it can be controlled with the help of sono-buoys. The FNs can freely move in the horizontal direction, but the movement in the vertical direction is negligible.
- All the sensor nodes, including the CHs, contain an equal amount of initial energies.
- The communication channel used is symmetric. This means that the amount of energy consumed by $N_{i}$ to forward data to $N_{j}$ is equal to the energy consumed by $N_{j}$ to convey its data to $N_{i}$ for a defined SNR.
- The FNs in the overlapping region contain the correlated data.
- FNs can perform sensing duties in sleep-mode or low-power listening mode, but they can communicate only in the active mode.

### 4.2. Setup Phase

This is first phase of HEEG that is divided into two parts. The network initialization and the neighbor discovery take place in this phase. The detail of this phase is discussed in the next subsection.

#### 4.2.1. Initialization

**Figure 2:**
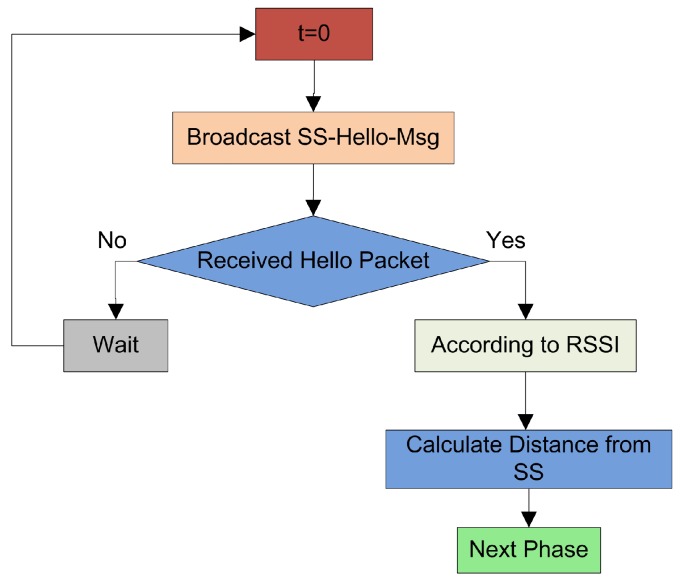
The flowchart of initialization of the proposed framework.

In this phase of the network, the FNs are, at random, deployed in the NSF. We take some important points into consideration like the FNs density and the communication distance during the installation to avoid the early exhaustion of the network, as the FNs installed in the NSF cannot be replaced or displaced. After the initialization, the SS broadcasts a SS−Hello−Msg and this message contains the coordinates of SS. After receiving this Hello message, all the FNs in the network update the location of the SS. The details of network initialization are shown in Figure 2.

#### 4.2.2. Neighbor Discovery

**Figure 3:**
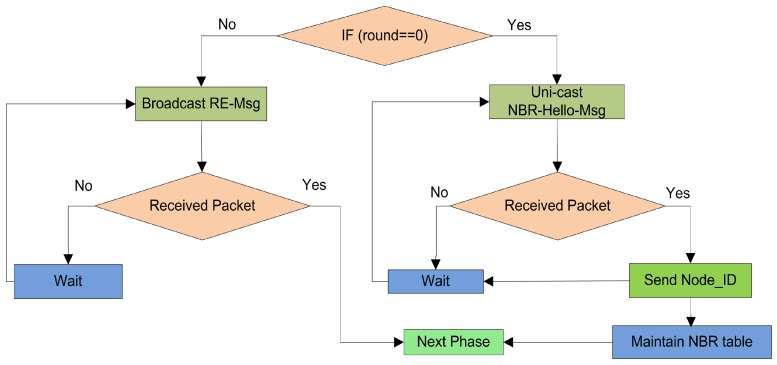
The pipeline of neighbor discovery.

The key purpose of this phase is to initially create a reliable infrastructure between the FNs for data exchange and without this infrastructure, it is not practically possible to exchange the control message. Every round starts up with the aim of upgrading the previous infrastructure. After this phase, the FNs become familiar with all the corresponding neighbors, their FN IDs and their location information. The FNs use the exchange of NBR−Hello−Message to inform the *1-hop* neighbor about the link status and the coordinates. The probability of communication between neighbor nodes increases with this phase, the particulars of this phase are shown in Figure 3.

### 4.3. Cluster Head Selection Game

The proposed model supposes that each FN has an adequate amount of energy and data to convey toward the SS. The CHs are only selected for the sake of energy preservation and only responsible for forwarding the data of SMNs to the next relay node towards the SS. All the alive nodes (FNs with sufficient amount of power level) exchange their messages with the neighbors in their communication range. Let NBR(i) express the number of neighbors in the communication radius of node *i* which can be expressed as:

(9) $$\begin{array}{c} NBR(i)=j|dis(i,j)\leq R,j\neq i \end{array}$$

where *R* expresses the communication radius and distance between the node *i* and node *j* is represented through dis(i,j). Each FN separately plays its Cluster-head Selection Game (CSG) with the neighbors and greedily chooses its rank for the existing round. For any FN *i*, its CSG can be determined using the following expression:

(10) $$\begin{array}{c} CSG=\langle N,S,U\rangle \end{array}$$

where *N* signifies the total number of players participating in the game including the FN *i* and its neighbors, while $S=\{S_{j}|j\left[N\right\}$ and $U=\{U_{j}|j\left[N\right\}$ indicates the player strategies and utility functions, respectively. All the FNs have two choices in each round like declaring its status as CHs and not declaring as CHs. If an FN selects a strategy then its payoff will be according to the selected strategy. If the FNs choose the strategy *D* its payoff will be *v*, which is equal to energy consumed for transmitting a data packet to the SS. If the player chooses a strategy ND its payoff will be a deducted amount of *e* that is the amount of overhead. The utility function for a node *i* can be expressed as:

(11) $$\begin{array}{c} U_{i}=\left\{\begin{array}{cc} v_{i}-e_{i} & if\;s_{i}=D\\ v_{i} & if\;s_{i}=ND\\ 0 & if\;s_{j}=D \end{array}\right. \end{array}$$

Letting $p_{i}$ be the probability that player *i* chooses the strategy *D*. According to Reference [10,11], a symmetrical mixed strategies Nash equilibrium exists, and the equilibrium probability $p_{i}$ is described as:

(12) $$\begin{array}{c} p_{i}=1-{\left(w\right)}^{\frac{1}{\left|Nb(i)\right|}} \end{array}$$

where, $v_{i}$ is the payoff for FN *i* on choosing the strategy ND when there exists at least one additional FN selecting the strategy *D*. However, $e_{i}$ is the extra cost for FN *i* on choosing the strategy *D* where w=e/v<1;|NBR(i)| is the number of elements in set NBR(i), which consists of the neighbors of players *i*.

#### 4.3.1. Equilibrium Strategy Calculation

**Figure 4:**
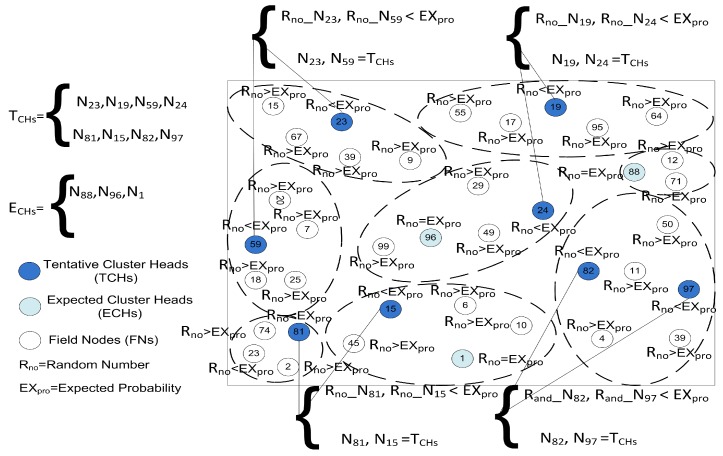
The tentative and expected CHs selection process of the proposed model on the basis of expected probability.

The CH selection process is an important step of our algorithm because it is performed in each round. This process is completed in two steps; in the first step, Tentative Cluster Heads (TCHs) are selected in the network and clusters are formed when the member nodes join these TCHs. In the second step, these selected TCHs are confirmed as Final Cluster Heads (FCHs) by limiting the number of member nodes to equally distribute the energy load on the network as shown in Figure 4. At the beginning of the network an expected probability $EX_{prob}$ is computed through the following equation:

(13) $$\begin{array}{c} EX_{prob}=\frac{F}{\pi {\left(\varepsilon R\right)}^{2}N} \end{array}$$

where, *F* and *R* are the NSF and communication radius, respectively, while N represents the number of FNs in the NSF and ε is a radius adjustment factor in the range of (0,1). After computing the $EX_{pro}$, each FN in the NSF generates a Random Number $R_{no}$ and determines its rank for the existing round by comparing $R_{no}$ with $EX_{pro}$. In comparing $R_{no}$ with $EX_{pro}$ there are the following cases:

- **Case 1:** If $R_{no}<EX_{pro}$, the FN will declare its status as TCHs for the existing round.
- **Case 2:** If $R_{no}>EX_{pro}$, the FN will remain the FN for the existing round.
- **Case 3:** If $R_{no}=EX_{pro}$, the FN will declare its status as Expected Cluster Head (ECH) for the existing round.

From the above-discussed cases, all the FNs will actively participate in this CSG and each TCH and ECH selected through $EX_{pro}$ can simply compute its next round equilibrium probabilities. Before moving further, we need to clarify that the number of TCH, ECH, and SMNs are equal to the total number of FNs. Second, the TCH acquired all the residual energies of the participating players and we also need to define the energy consumption per unit area according to Reference [30]. So, whenever a player *i* chooses a strategy ND and another player chooses strategy *D* their payoff can be calculated through the following equation:

(14) $$\begin{array}{c} v_{i}=\frac{k}{E_{cm_{i}}} \end{array}$$

where $E_{cm_{i}}$ is energy spent to convey k−bit data-packet to the concern TCH and computed using the following equation:

(15) $$\begin{array}{c} E_{cm_{i}}=kE_{elec}+kE_{amp}{\left(\frac{2}{3}R\right)}^{2} \end{array}$$

where 2R/3 expresses the average distance between FN and its corresponding CH. However, $e_{i}$ is the extra cost for FN *i* on choosing the strategy *D* can be calculated by:

(16) $$\begin{array}{c} e_{i}=v_{i}-\frac{k}{E_{ch_{i}}} \end{array}$$

where $v_{i}$ is the payoff for FN *i* on choosing the strategy ND when there exists at least one additional FN selecting the strategy *D*. $E_{ch_{i}}$ is used to express the energy expend by FN *i* in forwarding a data packet of *k* bits to the SS while acting as the FCH. The $E_{ch_{i}}$ can be computed using the following equation:

(17) $$\begin{array}{c} E_{ch_{i}}=kE_{elec}Nb_{i}+kE_{DA}\left(Nb_{i}+1\right)+kE_{amp}d_{i}^{4} \end{array}$$

Putting the value of $v_{i}$ as:

(18) $$\begin{array}{c} e_{i}=\frac{k}{E_{cm_{i}}}-\frac{k}{E_{ch_{i}}} \end{array}$$

(19) $$\begin{array}{c} \frac{e_{i}}{v_{i}}=1-\frac{E_{cm_{i}}}{E_{ch_{i}}} \end{array}$$

Let $w_{i}=e_{i}/v_{i}$, we have the following equation:

(20) $$\begin{array}{c} w_{i}=1-\frac{E_{cm_{i}}}{E_{ch_{i}}} \end{array}$$

In Equation (20), $E_{ch_{i}}$ is used to express the energy expended by FN *i* in forwarding a data packet of *k* bits to the SS while acting as the FCH. Sometimes $w_{i}$ acquires negative values; to avoid such a case, we add another variable $w_{min}$. Now, the above equation can be rewritten as:

(21) $$\begin{array}{c} w_{i}=\max\left(w_{\min},1-\frac{E_{cm_{i}}}{E_{ch_{i}}}\right) \end{array}$$

By using Equation (21) the equilibrium probability of any player *i* can be computed as follows:

(22) $$\begin{array}{c} EQ_{pro}=1-{\left(w_{i}\right)}^{\frac{1}{\left|Nb(i)\right|}} \end{array}$$

where, |Nb(i)| and $p_{i}$ express the neighbors FNs of player *i* and the average $EQ_{pro}$ of all the FNs inside the communication range *R* of the player *i*.

#### 4.3.2. Final CHs Selection

**Figure 5:**
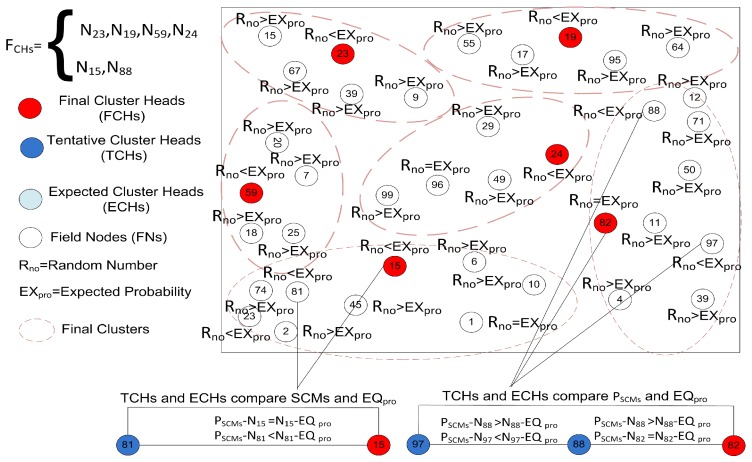
Finalizing the number of CHs from the TCHs and ECHs on the basis of equilibrium probability.

The network lifetime depends upon the energy management, controlled cluster size, and optimal number of CHs. If we control the cluster size and number of clusters in the network, the energy distribution will automatically optimize. To attain this energy goal, we optimize the number of TCHs through the $EX_{prob}$ and now we optimize the cluster size to reduce the redundant energy consumption. From the $EX_{pro}$ equation, we can note that each TCH is communicating at radius *r* with the SMNs. Consequently, every TCH and ECHs are covering an area equal to $\pi R^{2}$ in the NSF is $\frac{F}{\pi {\left(\varepsilon R\right)}^{2}N}$. So, here we need to verify that the selected number of expected TCHs and ECHs are completely covering the sensing field. For verification purposes, we used the $EQ_{pro}$ that helps us to select the number of TCHs and ECHs to meet the optimality criteria for cluster size as shown in Figure 5. Each TCH ensures its rank as a FCH in the existing round by comparing selected member probability $P_{SCM}$ with $EQ_{pro}$. Each TCH and ECH will compute the $P_{SCM}$ using the following expression:

(23) $$\begin{array}{c} P_{SCM}=1-{\left(w_{i}\right)}^{\frac{1}{\left|SCM(i)\right|}} \end{array}$$

On comparing the $P_{SCM}$ with $EQ_{pro}$, we have the following three cases:

- **Case 1:** If $P_{SCM}<EQ_{pro}$, the TCHs will add more CMs in the cluster until reaching the $EQ_{pro}$. The ECHs also repeat the same process and if they cannot attain the $EQ_{pro}$ state, these ECHs quit from the current status and become the FNs.
- **Case 2:** If $P_{SCM}>EQ_{pro}$, the TCHs will cancel some SCMs in the cluster until reaching the $EQ_{pro}$. The ECHs also repeat the same process and if they cannot attain the $EQ_{pro}$ state, these ECHs quit from the current status and become the FNs.
- **Case 3:** If $P_{SCM}=EQ_{pro}$, the TCH will declare its status as an FCH for the existing round.

After the successful completion of the FCH selection phase, the TDMA slot allocation starts. The FCH selects nodes from a different direction and assigns them the TDMA slots. For this selection, only those nodes are chosen that are not in overlapping range of other nodes. The nodes that do not perform the sensing activity remain in sleep mode to save the available resources. While the chosen nodes start sensing their fields, at the arrival of their time slots these nodes send their data to their corresponding FCHs. There is no need for time scheduling to forward the data to the SS. FCHs can forward the data to their corresponding SS anytime during a round. The SCH collects the data from the SMNs in the cluster and forwards it to the next FCHs toward the SS. The relay selection criteria are discussed in the next subsection.

### 4.4. Relay Selection Game

In the non-cooperative evolutionary relay selection game, each player separately plays its relay selection game with the neighbors and greedily chooses its strategy for the existing round. For any FN *i*, its relay selection game can be determined using the following expression:

(24) $$\begin{array}{c} RSG=\langle N,S,U\rangle \end{array}$$

where, *R* and $S=\{s_{r}|r\in R\}$ express the set of available hops and strategy space for the players, respectively. When the players choose strategies like $s_{r}$ and $s_{t}$ their payoff is indicated through $u(s_{r},s_{t})\in U$. In this evolutionary relay selection game, we preferred the low communication cost which results in the higher payoff and helps in saving the system energy. The utility function is expressed as:

(25) $$\begin{array}{c} U\left(s_{i},s_{j}\right)=\left\{\begin{array}{cc} \left(\frac{1}{c_{i}},\frac{1}{c_{j}}\right) & if\;i\neq j\\ 0 & if\;i=j \end{array}\right. \end{array}$$

where, $c_{r}$ represents the communication cost for forwarding data through *r* hops. Now we present the fitness for the replicator dynamics; for this purpose we consider two randomly paired players. The players have already assumed the set of hops *H* and strategies $s_{h}\in S$. Suppose that $\overset{`}{p}\;=\;\left\{p_{1},\;p_{2},p_{3},\ldots ,p_{r}\right\}$ and $\overset{`}{q}=\left\{q_{1},q_{2},q_{3},\ldots ,q_{h}\right\}$ signify the proportion of the other players approving $s_{1},s_{2},s_{3},\ldots ,s_{h}$ strategies, respectively, where the summation of the proportions is equal to 1 (i.e., $\sum_{i=1}^{h}p_{i}=1\;and\;\sum_{i=1}^{h}q_{i}=1$). Let $p_{h}$ and $q_{h}$ be probabilities for choosing the next hops ($\overset{`}{p}\;and\;\overset{`}{q}$), respectively. Furthermore, $U=\{u_{1},u_{2},u_{3},\ldots u_{h}\}$ and $u_{h}$ denote the average payoff and utility function for choosing $s_{h}$ strategy. The payoff for adopting $s_{h}$ strategy for player (A) can be expressed as:

(26) $$\begin{array}{c} u_{i}=u_{o}+\sum_{g=1}^{\left|R\right|}p_{h}u\left(s_{h},s_{g}\right)\;\;\;\;\;\;\;\;\;\;\forall h,g\in ℜ \end{array}$$

The payoff for adopting $s_{g}$ strategy for player (B) can be expressed as:

(27) $$\begin{array}{c} u_{i}=u_{o}+\sum_{g=1}^{\left|R\right|}q_{g}u\left(s_{h},s_{g}\right)\;\;\;\;\;\;\;\;\;\;\forall h,g\in ℜ \end{array}$$

where, $u_{0}$ and $u(s_{h},s_{g})$ express the initial fitness and fitness for adopting *h* hop against *g* hop. Let $u_{A}$ be the average fitness for player A and $u_{B}$ be the average fitness for player B, expressed through the following notations:

(28) $$\begin{array}{c} \bar{u_{A}}=\sum_{m=1}^{i}q_{m}u\left(p_{m}u_{m}\right)\;\;\;\;\;\;\;\;\;\;\forall m\in ℜ \end{array}$$

For every next slot, the probability $\left({\overset{ˇ}{p}}_{h},{\overset{ˇ}{q}}_{h}\right)$ for adopting the next hop in our relay selection game is computed as:

(29) $$\begin{array}{c} \bar{u_{B}}=\sum_{m=1}^{i}p_{m}u\left(q_{m}u_{m}\right)\;\;\;\;\;\;\;\;\;\;\forall m\in ℜ \end{array}$$

The proportion of FNs adopting *h* hop in the next TDMA slot can be increased or decreased depending upon the comparison of overall fitness to the average fitness for the entire network in the existing TDMA slot. The frequency of the next hop selection will be increased if the payoff is higher than the average fitness for the entire network.

(30) $$\begin{array}{c} {\overset{ˇ}{p}}_{h}=p_{h}+\frac{q_{h}(u_{h}-\bar{u_{A}})}{\bar{u_{A}}} \end{array}$$

(31) $$\begin{array}{c} {\overset{ˇ}{q}}_{h}=q_{h}+\frac{p_{h}(u_{h}-\bar{u_{B}})}{\bar{u_{B}}} \end{array}$$

#### Treating with Floated FNs

**Figure 6:**
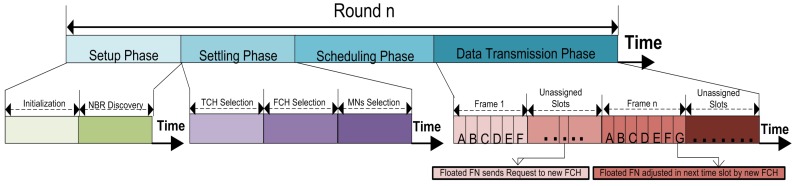
The description of floated FNs adjustment due to water current movements.

Currently, in clustering-based structure the FNs convey data through CH instead of forwarding the data individually; a head node is chosen for forwarding the sensed information of all the member nodes [15]. The head node performs some extra duties like data collection and data fusion [13,15,16]. However, due to the poor selection criteria or random selection CH, some CHs are chosen without any SMNs. These CH forward own data in the entire round, this redundant data is an overall loss of energy resources. Secondly, due to the changing underwater conditions, the nodes constantly move with the water currents. Therefore, the node’s movements create difficulty in managing the communication links and capturing the exact location of forwarder nodes. The proposed model defines a clear strategy to overcome the problem of node movement and self-selected CHs. These nodes can convey the data through the nearest SMN or FCH. The nodes floated in any cluster will be treated as an SMN after sending the request to the new FCH. The detailed description of the floated nodes adjustment is given in Figure 6.

## 5. Performance Evaluation

In this section, we perform simulations to analyze the performance of the proposed framework HEEG using ns-3 featuring the underwater model developed in Reference [32]. In the simulation, we use the underwater acoustic channel ns3 :: Uanchannel, MAC layer protocol CW−MAC802.11DCF, packet error rate model ns3 :: UanphyperGendefault, and underwater traversing is used to identify the network link breakage as defined in Reference [32]. We compare our framework with three state-of-the-art schemes: ADDP [20], PRADD [21] and DEEP [14]. The schemes we compare with our method are chosen due to these reasons: (1) citations; (2) recency; (3) dealing with the same problem in a different way; and (4) availability of the solution. For a fair comparison, the same simulation environment and the same parameters are used for all these approaches. Average results included in these simulations have a 90% confidence interval, which is acquired after running the simulation 5 times. The parameters used for simulation purpose are elaborated in Table 1.

### 5.1. Analysis of Our Framework

**Figure 7:**
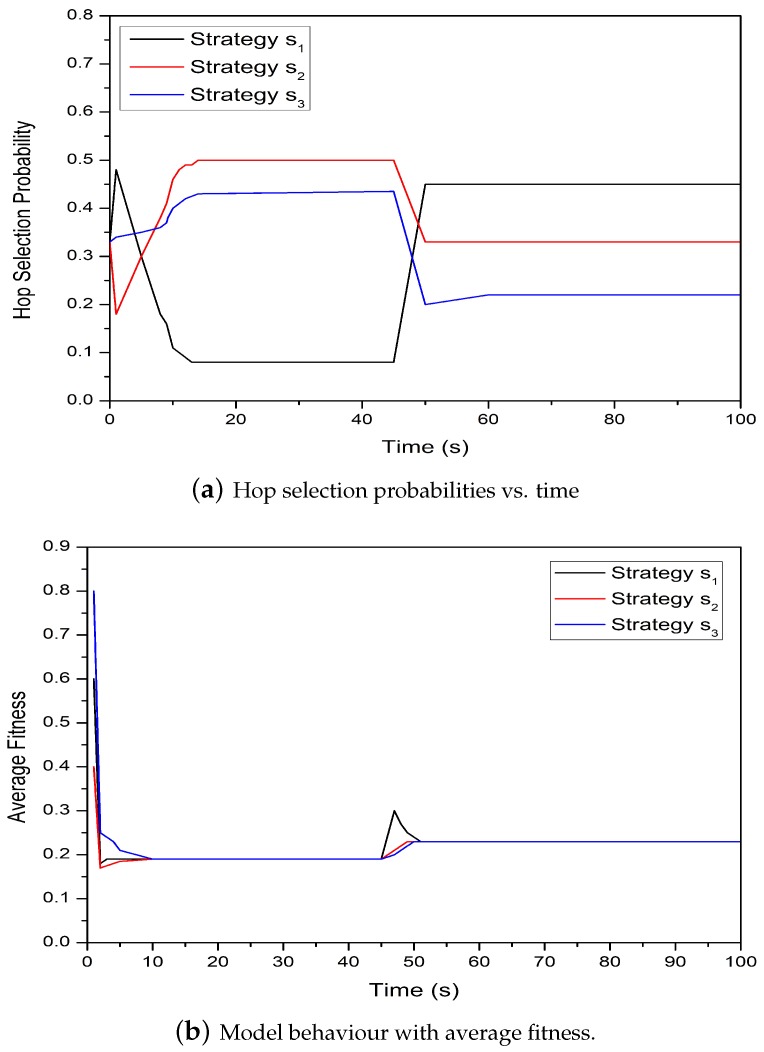
The comparison of average fitness and hop selection probability with time for the proposed model.

Figure 7a demonstrates the behavior of the FNs for hop selection probabilities and time with changing network conditions. From the Figure 7a, we can note that initially the model behaves differently at equal and unequal probabilities and time=45. Adopting any strategy at time $s_{1}$ is more attractive and with a higher payoff as compared to strategies at times $s_{2}$ and $s_{3}$, respectively. Figure 7b shows the comparison of average fitness and time for the proposed model. From the graph, we can note that the convergence probabilities for selecting the next hop are totally dependent upon the average fitness function. These probabilities can also be modified according to the average fitness and this modification can be gained through strategies interacting in a succeeding time slot.

### 5.2. Data Delivery Ratio

**Figure 8:**
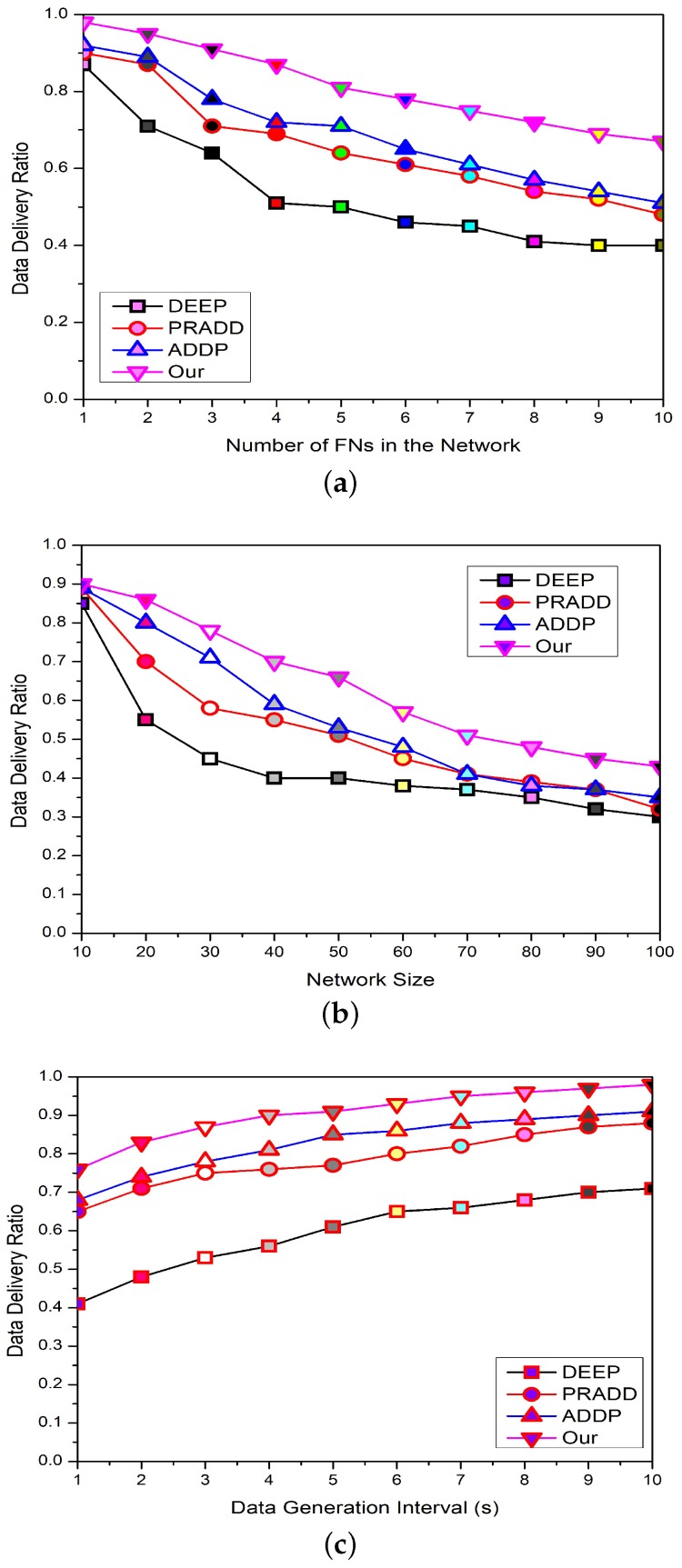
The comparison of data delivery ratio of our proposed framework with different values of number of field nodes, network size, and data generation interval.

The packet delivery rate is directly related to the data collision and the propagation link status between the FNs and the FCHs. The multi-hop communication between FNs can increase the chances of data collision. Similarly, if the propagation link status is not good enough, then the data will not successfully deliver at the SS. Figure 8a depicts the comparison of the packet delivery ratio with the increasing number of FNs in the network among the proposed models—ADDP [20], PRADD [21], and DEEP [14]. From the graph, it is obvious that increasing the number of FNs in the network can increase the generated packets. The packet delivery ratio of the proposed framework is greater than other models because the proposed scheme does not use any greedy strategy for the forwarder selection. The nodes at optimal routes remain busy for forwarding the data of their predecessors. The multiple nodes are trying to send the data through the optimal route and drop the packet after many tries. The optimal route selection is valid up to a few hops but as the number of hops increases, there are fewer chances for a packet to be delivered successfully. The proposed model has a good delivery ratio because the next hop selection for forwarding a data packets depends upon the available, not on the optimal routes. As a result, there are very fewer chances for data packets to be dropped. Last but not least, if a node with a data packet moved into the neighboring cluster due to water currents movements, it can easily forward the data according to the assigned schedule. The FNs can adjust themselves according to the water current movements, so the FNs mobility pattern cannot seriously affect its delivery ratio.

Figure 8b depicts the comparison of the packet delivery ratio with the increasing size of the network between HEEG, ADDP [20], PRADD [21], and DEEP [14]. The packet delivery ratio of the HEEG is greater than that of the other models because its data forwarding hierarchy is consistent as it selects the congestion-free routes instead of available routes for data delivery. So, HEEG has better adjustment capacity for route selection as compared to ADDP [20], PRADD [21], and DEEP [14]. As the network size increases, the large number of nodes try to send the data through the optimal route; as a result, some packets may be lost after many tries. The optimal route selection is valid up to a few hops but as the number of hops increases, there are fewer chances for a packet to be delivered successfully. The CHs closer to the SS are always busy forwarding the data of their predecessors due to heavy traffic load. These CHs have the same capabilities as other FNs, so most of the data is lost here due to congestion and heavy traffic load.

Figure 8c portrays the comparison of the packet delivery ratio with increasing the data generation interval between HEEG, ADDP [20], PRADD [21], and DEEP [14]. From Figure 8c, we can see that with an increasing data generation interval, the chances of data collision are decreased. Consequently, maximum packets are successfully delivered at the destination. ADDP has a lower packet delivery ratio because in this protocol the data cannot be conveyed to the next hop until the data packet has reached its maximum packet size. Most of the time, the packet is discarded when the maximum packet size cannot be attained within the defined time limits. In the case of PRADD and DEEP, the data relaying gateways cannot hold a packet for a long time and hand it over to the neighbors to forward it. So, data travel takes longer as compared to the average time and some time is lost due to the route mismanagement, which affects the packet delivery ratio of these protocols.

### 5.3. Network Energy Consumption

**Figure 9:**
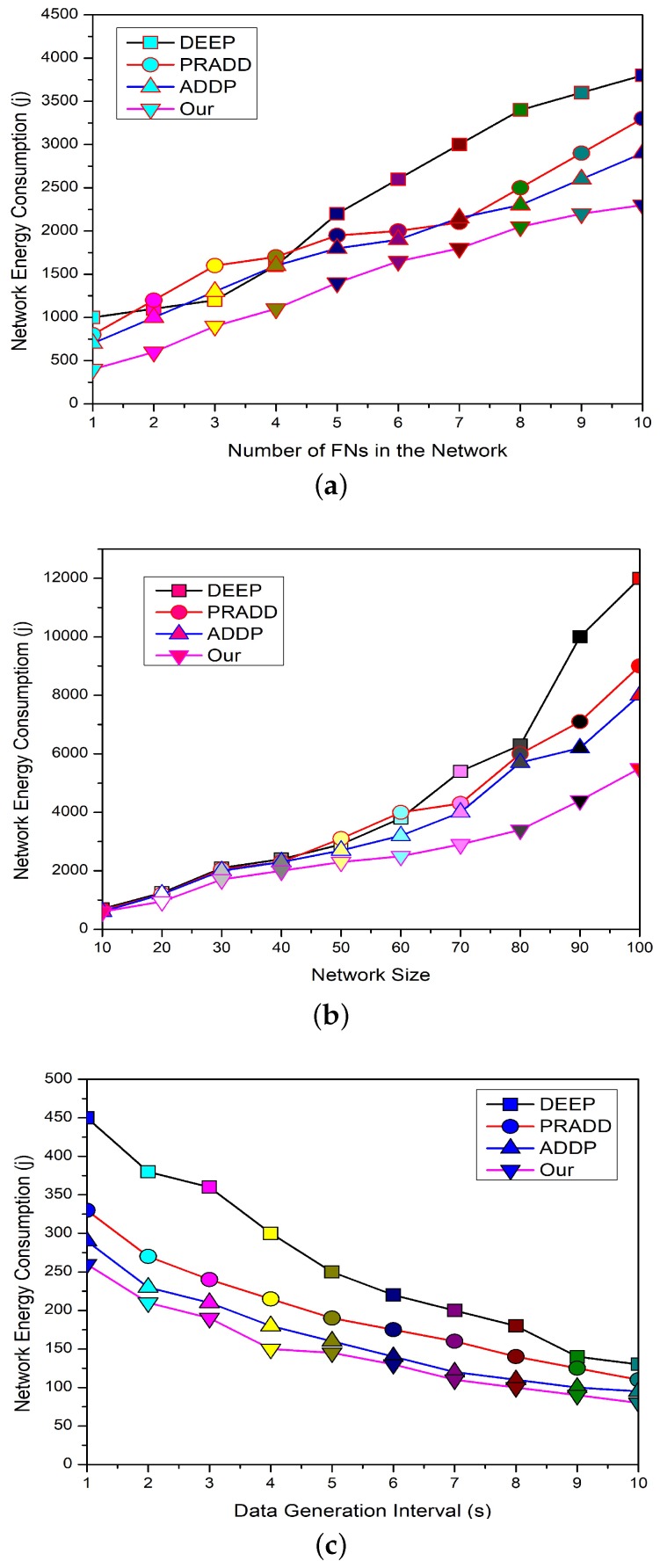
The effect of number of field nodes, network size, and data generation interval on the network energy consumption of the proposed model.

Figure 9a elaborates the comparison of energy consumption and the number of FNs in the network between ADDP [20], PRADD [21], and DEEP [14]. The presented results show that the proposed framework achieves a longer lifetime with less energy consumption as compared to the counterpart schemes. In the proposed model, the energy load on each node is balanced in a distributed manner. The FNs in the proposed model are forced to use the single-hop as well as the multi-hop transmission ranges to communicate with CHs depending on the situation. HEEG controls the repeated transmission by selecting a few nodes to perform the sensing activity in the overlapping region. The CHs are redundancy-aware in HEEG, so only selected information can travel toward the SS to save the available energy of the network. The counterpart schemes do not define a clear strategy to overcome the issues of redundant data and repeated information.

Figure 9b demonstrates the comparison of energy consumption and the network size between ADDP [20], PRADD [21] and DEEP [14]. From the graph, we can see that the proposed framework has less energy consumption as compared to the counterpart schemes. The proposed model has strong CHs management, so increasing the size of the network cannot affect the network energy consumption. While in the case of ADDP, PRADD and DEEP the increasing network size cannot affect energy consumption to some extent, if we keep increasing the number of nodes these schemes face some serious problems.

Figure 9c shows the comparison of energy consumption and data generation interval between ADDP [20], PRADD [21] and DEEP [14]. From the graph, we can see that the proposed framework achieves a longer lifetime with less energy consumption as compared to the counterpart schemes. In the case of ADDP, as the data generation interval increases the more AUVs move in the network to receive the same data, which increases the network energy consumption. This is the same in the case of PRADD; the ferry moves more frequently around the network to collect the data to attain the maximum data limit, which increases the energy consumption. In the case of DEEP, the data generation interval increases the energy consumption of the network because the data travels a long way to avoid collisions.

### 5.4. Routing Overhead

**Figure 10:**
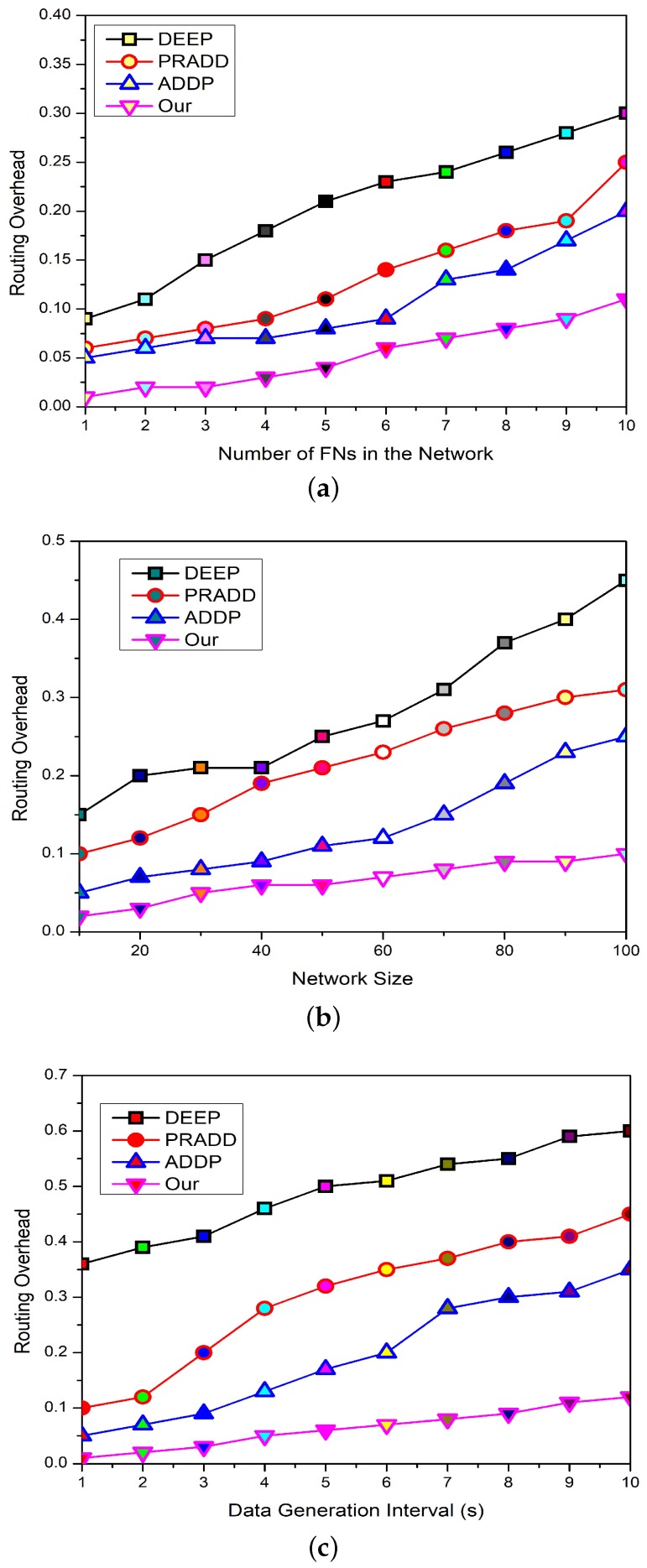
The comparison of routing overhead with number of field nodes, network size, and data generation interval.

Figure 10a depicts the comparison of the routing overhead with increasing the number of FNs in the network between the proposed model, ADDP [20], PRADD [21], and DEEP [14]. The overhead of the proposed framework is less than other models because in the proposed scheme, the data forwarding hierarchy remains the same and the burden on CH increases with increasing number of nodes. However, the selected information travels inside the network, which controls the routing overhead and stabilizes the network. ADDP has less overhead as compared to PRADD and DEEP because an ad-hoc on-demand connection is established for forwarding the data. So, more and more nodes are trying to establish the connections with their neighbors, as a result, the overhead increases. While in the case of PRADD and DEEP, as the number of nodes increases the more routing overhead is generated in the network due to connection and disconnection between the FNs and the gateways. From the graph, it is obvious that DEEP suffers more as compared to the opponent’s schemes because the optimal and collision-free routes are selected on the cost of routing overhead.

Figure 10b depicts the comparison of the routing overhead with the network size between the proposed model, ADDP [20], PRADD [21], and DEEP [14]. The overhead of the proposed framework is less than that of the other models because the proposed scheme data forwarding hierarchy is simple, so it is less affected by the increased network size. ADDP has less overhead as compared to PRADD and DEEP because only the data forwarding overhead is increased with increasing the size of the network. While in the case of PRADD and DEEP, as the number of hops increases, the more control overhead is generated, which affects the network efficiency.

Figure 10c shows the comparison between the data generation interval and the routing overhead of ADDP [20], PRADD [21], and DEEP [14]. The increasing data generation interval does not increase the routing overhead in HEEG as compared to the counterpart schemes, while the ADDP, PRADD, and DEEP lifetime and efficiency are seriously affected with the longer data interval as compared to the HEEG. This is because with the longer data generation interval the gap between data packets becomes longer and the relay nodes start forwarding the data of individual FNs without fusing it as shown in Figure 10c.

## 6. Conclusions

In this paper, we have introduced a novel hybrid energy equating game for monitoring the harsh underwater environment with less energy consumption. We propose a cooperative clustering game that is based upon energy heterogeneity and a penalty mechanism. All the FNs actively participate in this cluster selection game and each CH selected through expected probability can compute its next round equilibrium probability. As a result of this clustering game, only FNs with higher energy and the minimum payoff are selected as CHs. Then, we propose a non-cooperative evolutionary game for the next hop selection in which each player separately plays its relay selection game with the neighbors and greedily chooses its strategy for the existing round. This next hop strategy is not invaded by any other mixed strategies and the nodes can modify their strategy until they reach the stable state (destination). We also prove that this utility function for an evolutionary relay selection game is the best suitable solution for the forwarder selection. The schemes selected for comparison with the proposed model are recent in the literature and deal with the same issues in different ways. The proposed framework is evaluated against recent schemes using different quality measures and we found that our proposed framework performs favorably against the recent schemes for all of the evaluation metrics. In future work, the lifetime of the HEEG can be further enhanced by taking into consideration energy harvesting. We are also planning to study clustering with different renowned optimization algorithms and preparing to include the mobile CHs for data collection. 

## Figures and Tables

**Table 1:** Parameter values used for the simulations.

Variable	Value
Number of field nodes	100–800
Network area	800 × 800 × 800 m
Speed of sound	1500 m/s
r	10–50 m
Transmission range	250 m
Transmit power	50 W
Bandwidth	80 Hz
Width of layer	200 m
$E_{int}$	2000 J
$E_{elec}$	50 nJ/bit
A(f)	1.001
$d_{0}$	80–100 m
$E_{da}$	50 nJ/bit/packet
Data rate	5 Kb/s
Data packet size	64 bytes
Header size	13 bytes
Nodes mobility	1–5 m/s
Acoustic pressure of layer	101 dB
Acoustic pressure of data transmission	103 dB
Total run time	1000 s

## Abbreviations

The following abbreviations are used in this manuscript:

IoUTs	Internet of Underwater Things
GT	Game Theory
CH	Cluster Head
NSF	Network Sensing Field
AUV	Autonomous Underwater Vehicle
HEEG	Hybrid Energy Equating Game
ADDP	AUV-based Data Delivery Protocol
DEEP	Delay-aware Energy Efficient Protocol
PRADD	Path Reliability-Aware Data Delivery
MS	Mobile Sink
SS	Surface Sink
FNs	Field Nodes
SMNs	Selected Member Nodes
FCH	Final Cluster Head
CSG	Cluster-head Selection Game
TCHs	Tentative Cluster Heads
ECHs	Elected Cluster Heads
RSG	Relay Selection Game
